# Influence of Particle Rotation on the Shear Characteristics of Calcareous-Sand and Silica-Bead Granular Materials

**DOI:** 10.3390/ma17235827

**Published:** 2024-11-27

**Authors:** Tao Li, Jiajun Shu, Yuming Wu, Yue Li, Bingni Wu, Zhengding Deng, Jingzhu Huang, Rubén Galindo, Fausto Molina Gómez

**Affiliations:** 1 Inner Mongolia Research Institute, China University of Mining and Technology-Beijing, Ordos 017000, China; litcumt@163.com; 2 School of Mechanics and Civil Engineering, China University of Mining and Technology-Beijing, Beijing 100083, China; 3 Higher Technical School of Civil Engineers, Technical University of Madrid, 28039 Madrid, Spainfausto.molina@upm.es (F.M.G.); 4 Jiangxi Province Key Laboratory of Environmental Geotechnical and Engineering Hazards Control, Jiangxi University of Science and Technology, Ganzhou 341000, China; 5 School of Civil Engineering and Architecture, East China Jiaotong University, Nanchang 330013, China

**Keywords:** calcareous sand, particle rotation, shape parameters, energy evolution, shear characteristics

## Abstract

The shear strength and resistance of granular materials are critical indicators in geotechnical engineering and infrastructure construction. Both sliding and rotation influence the energy evolution of soil granular motion during shear. To examine the effects of particle rotation on shear damage and energy evolution in granular systems, we first describe the transformation of irregularly shaped particles into regular shapes via geometrical parameters, ensuring the invariance of energy density and density. We then analyze the impact of particle rotation on shear-stress variation and energy dissipation through a shear energy evolution equation. Additionally, we establish the relationship between the shear-stress ratio and normal stress, considering particle rotation. Finally, we verify the influence of particle rotation on energy evolution and shear damage through shear tests on irregular calcareous sand and regular silica-bead particles. The results indicate that granular materials do not fully comply with the Coulomb strength criterion. In the initial shear stage, most of the external work is converted into granular rotational-shear energy, whereas in the later stage, it primarily shifts to granular sliding-shear energy. Notably, the sensitivity of the granular rotational energy to a vertical load is significantly greater than that of the granular sliding energy.

## 1. Introduction

Almost all human engineering and transportation infrastructure depends on geotechnical bodies, which are typically composed of granular materials, such as rockfill dams, dam foundations, roadbeds, tunnels, and load-bearing layers of buildings or structures. Owing to the complex and diverse mechanical properties of granular materials, many researchers have studied these properties in depth, including phenomena such as acoustic fluidization, shear thickening, non-homogeneity, and anisotropy [1,2,3,4]. Among these studies, those focused on the shear properties of soil granular materials under different engineering conditions are of particular interest, given the significant impact of shear properties on the deformation and failure characteristics of these materials [5,6,7,8]. Soil granular materials are highly heterogeneous, multifield, and multiphase media composed of discrete or cemented mineral particles and pore-filling fluids or gases. The internal structural evolution of these materials varies significantly under different dynamic loading conditions, resulting in physico-mechanical properties that differ from those of continuous media. Since granular particle systems are typically multiscale, with multiple levels of physical structure and characteristic time scales, the mechanical properties of soil particles are influenced by microstructural and geometrical shape parameters. These, in turn, manifest at various scales, including the microparticle scale, delicate microfabric scale (which characterizes bulk metrics), and macroscopic unit scale.

Calcareous sands are widely distributed in waters near the equator and along major coastlines worldwide, and engineering projects related to these sands are becoming increasingly common, while seismic activity and human interventions in offshore regions continue to be studied. However, these sites face significant risks, including seismic liquefaction and shear failure [9,10,11,12]. The South China Sea, in particular, holds vast deposits of calcareous sand, and owing to its abundant supply, the “winch suction blowing and filling” technique has become the primary method for artificial island foundation construction. With the advancement of China’s ocean development strategy and the “Maritime Silk Road” initiative, island and reef construction has emerged as a key strategic focus. The South China Sea is situated at the intersection of the Eurasian, Pacific, and Indian plates, close to the Philippine subduction zone, making the region highly susceptible to earthquakes and wave impacts. Consequently, future island and reef projects in the area must contend with the potential for strong seismic events and wave activity. Ensuring the stability of “blowfill” sites on these islands and reefs is critical for project safety. Calcareous-sand particles, along with the soils formed by blowfill processes, exhibit unique properties, including irregular particle shapes, high porosity, compressibility, low strength, fragility, and internal pores. These characteristics make calcareous sands markedly different from the land-based cohesionless soils typically used in engineering. There are currently many challenges in the engineering application of calcareous sands. For example, Yuan et al. [13] concluded that these sands are resistant to liquefaction, or are difficult to liquefy, as it is challenging to replicate the typical liquefaction phenomena of coral sands, such as saturated sandy soils, under laboratory conditions. Similarly, He et al. [14] reported that the anisotropic nature of calcareous sands significantly influences their physicomechanical properties, further complicating the use of these sands in construction.

In contrast to continuous media, the analysis of granular calcareous-sand materials focuses more on individual discrete particles rather than the entire system [15,16,17,18]. Under prolonged external loading, changes in the structural and constitutive properties of individual particles can lead to variations in the physical and mechanical properties of the overall material [19,20]. To investigate the direct effects of loading on individual particles, researchers have employed various methods, such as mechanical tests and numerical simulations, to examine the macroscopic mechanical properties of different granular materials. For example, Katarzyna [21] applied friction state theory and direct shear tests to determine the stress–expansion relationship for both large and small shear cases. Similarly, Sun et al. [22] analyzed the expansion and crushing-energy dissipation patterns of various particles via energy equations and reported that breaking energy dissipation increased significantly during the initial loading stage and stabilized over time, with the dissipation ratio between breaking energy and plastic work remaining nearly unaffected by peripheral pressure. Tian et al. [23] used the discrete element method (DEM) to simulate variations in shear zone thickness across different particle shapes and developed an empirical formula to describe the relationship between particle stress and shear strain. With the advancement of micromechanics and microscopic scanning technologies, there has been a notable increase in the number of studies on fine particle properties, focusing primarily on aspects such as particle geometry, cementation strength, and movement patterns [24,25,26,27,28,29,30]. However, the analyses mentioned above typically focus on the relationship between the microscale properties of particles and the macroscopic mechanics of the system from an image-based perspective, which fails to capture the kinematic behavior of individual particles and their role in shear damage. In the shear-damage analysis of granular materials, most existing studies assume that granules are ideal, circular discrete media and that slippage under shear force is the primary cause of damage [31,32,33]. In reality, the shapes of calcareous-sand particles vary significantly, and treating arbitrarily shaped particles as ideal round granules can lead to inaccuracies in energy and density calculations for the system [34]. Additionally, particles exhibit both rotational and slip behavior when subjected to shear stresses, and neglecting particle rotation while focusing solely on slippage alters the energy dissipation history of the granular system [35].

In summary, many studies have focused on exploring the effects of particle shape on the shear properties of granular materials through traditional methods (e.g., fabric evolution), whereas relatively few studies have investigated the effects of particle shape from other perspectives and methods. On the other hand, although studies have attempted to establish a link between the microstructure and the macroscopic mechanical behavior of granular materials, most of these studies are limited to the analysis of round or spherical particles. The influence of particle shape on the mechanical properties of geotechnical materials still needs to be explored in depth, because the study of different particle shapes in geotechnical materials is still insufficient. For this purpose, we introduced parameters related to the shape of the particles and analyzed irregular particles by transforming them into regular shapes under specific conditions. On this basis, we quantitatively analyzed the energy dissipation laws associated with the particle shape and rotational behavior of two types of materials, relatively irregular calcareous sand and relatively regular glass beads, through indoor shear tests. The results reveal the specific contributions of rotational and sliding energies throughout the shear disruption process and elucidate how particle shape affects particle rotational behavior at the mechanistic level. These findings further reveal the essential differences between granular earth materials and continuous media during shear damage.

## 2. Effect of the Particle Shape on the Rotational Characteristics

Under long-term geological and weathering conditions, calcareous-sand particles develop random, irregular shapes. At the microscopic scale, the geometry of calcareous-sand particles differs significantly from the ideal circular shape commonly assumed in many studies. These substantial geometric differences between calcareous sand and silica-bead particles significantly impact their kinematic properties and energy evolution. To investigate the multiscale shape characteristics of these particles, it is essential to define several parameters that can accurately describe their shapes. Yang et al. [36] employed laser scanning analysis to determine particle shapes in a two-dimensional plane. Subsequent studies have confirmed the reliability and applicability of these shape parameters, showing that they are well suited for quantitatively characterizing the geometry of particles of earth [37,38].

**Figure 1:**
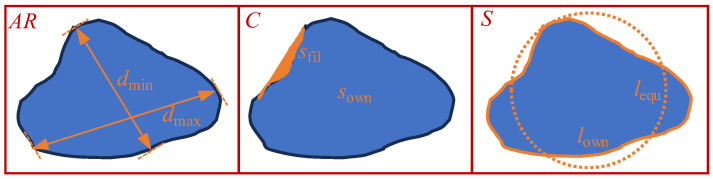
Schematic characterization of the shape parameters of granular materials.

To minimize the influence of the particle shape on the particle rotation process, a quantitative description of the particle shape, using shape-related parameters, is needed. To quantify the shape parameters objectively and accurately, we integrated shape-related parameters such as the aspect ratio (*AR*), convexity (*C*), and sphericity (*S*) of the granular materials, where *AR* is the ratio of the minimum Feret diameter *d*_min_ to the maximum Feret diameter *d*_max_ of the particle, which is considered the fine length of the material. Feret diameter is a key parameter in particle size analysis and is defined as the distance between any two points on the projected contour of a particle in a given direction. The line of this distance must be parallel to the specified direction and must completely encompass the projected contour of the particle. Ferrit diameters are categorized into minimum and maximum diameters. The *d*_min_ is the minimum distance between any two points in all possible directions on the projected contour of the particle, whereas *d*_max_ is the maximum distance between any two points in all possible directions on the same contour. *C* is the ratio of the area of the particle itself, *s*_own_, to its filled area (*s*_own_ + *s*_fil_), which can be considered the degree of convexity of the materials. *S* is the ratio of the equal-area circle circumference of the particle to its own circumference, which can be regarded as the degree of difference between the material and the ideal circle. *AR*, *C*, and *S* are shown schematically in Figure 1.

According to the calculation methods proposed by Yang et al. [36] and Altuhafi et al. [39], we can obtain the mathematical expressions of *AR*, *C*, and *S*, as shown in Equation (1).

(1) $$AR=\frac{d_{\min}}{d_{\max}},\;C=\frac{s_{\mathrm{own}}}{s_{\mathrm{own}}+s_{\mathrm{fil}}},\;S=\frac{l_{\mathrm{equ}}}{l_{\mathrm{own}}}$$

Each of the three parameters characterizing the particle geometry controls a different property of the particle shape; namely, *AR* controls the narrow or stubby property of the particle shape, *C* controls the concave property of the particle, and *S* controls the property of the gap between the particle shape and the standard circle. We know that the information describing the shape of the particles cannot be described by a single parameter, and another shape parameter can be given by weighting the three parameters *AR*, *C*, and *S*.

At present, detailed information about the particle shape should not be described by only one or two parameters, and the weighting coefficients of these three parameters are not yet clear. Therefore, Yang et al. [36] proposed a new shape measurement, namely, the overall regularity (*OR*), which is defined by combining the average values of the three shape parameters *AR*, *C*, and *S*, as shown in Equation (2).

(2) $$OR=\frac{1}{3}\left(AR+C+S\right)$$

**Figure 2:**
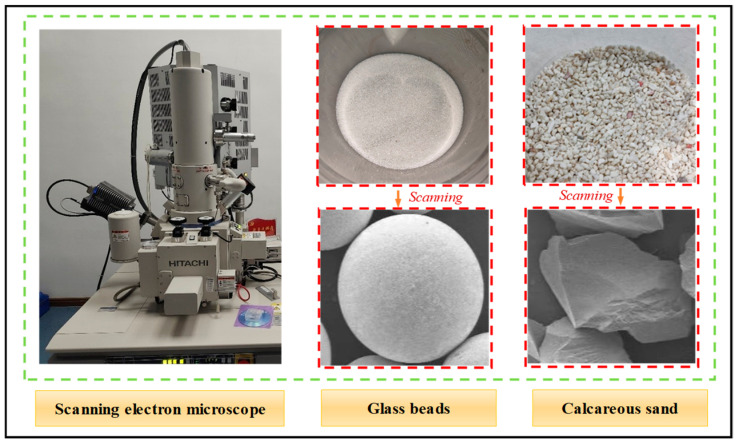
Electron microscopy images of the two granular materials.

With the shape parameter defined above, the *OR* takes values ranging from 0 to 1, which means that the *OR* value for an ideal round particle is 1. To analyze the shape parameters, we selected irregular calcareous material from an island in the South China Sea area as the research object. Additionally, relatively regular silica glass beads were selected for comparison. We obtained planar images of these two materials via an electron microscope scanning system, from which representative particles were selected for shape scanning and parameter calculation. Owing to the significant differences in the shapes of the spherical regular silica glass beads and irregular calcareous-sand particles, we obtained micro-CT images of the two particulate materials through electron microscope scanning experiments to characterize their morphology in detail. The micro-CT images were subsequently processed by image analysis software to determine the morphological parameters of the particles. The electron scanning electron microscope is model SU-8010 manufactured by HITACHI in Japan. The scanning results are detailed in Figure 2.

Four parameters, *AR*, *C*, *S*, and *OR*, were calculated for individual glass beads and calcareous-sand particles via Equations (1) and (2). However, it is important to note that the above calculations involve only individual particles, and the results are subject to greater randomness and chance. To increase the reliability of the results, we identified and analyzed all the particles in the electron microscope scanning images via image analysis software, and the results were collected and averaged according to the identification results. On the basis of the results of these analyses, we obtained the average shape parameters of the glass beads and calcareous sand, and the results are shown in Table 1.

In Table 1, Typ denotes the calculated results for a set of materials, Avg denotes the average value determined based on the materials in a standard sample, and Range denotes the range of values determined based on the particles in a standard sample. According to the data in Table 1, regular glass beads have ORs close to 1, whereas irregular calcareous sand has ORs far from 1. Therefore, treating some kinds of particles (e.g., calcareous-sand materials, as in this paper) directly as round particles in granular material analysis has several limitations and drawbacks [37]. Moreover, observing the calcareous-sand particles in Figure 2 reveals that it is more appropriate to use ellipses to describe the particle shape of calcareous sand than to use circles, but the use of ellipses increases the complexity of the analysis when the rotational characteristics of the particles are considered. To overcome the limitations of direct assumptions and the complexity of utilizing elliptic analysis, we consider a conditional shape transformation model for granular materials. First, the elliptic function most similar to the shape of the particle is constructed by the mapping function; second, the circular function equivalent to the elliptic function is constructed by the particle kinematic properties; and finally, the transformed circular function is utilized to simulate the rotational properties of the particles to analyze the shear-damage characteristics of the granular materials.

**Figure 3:**
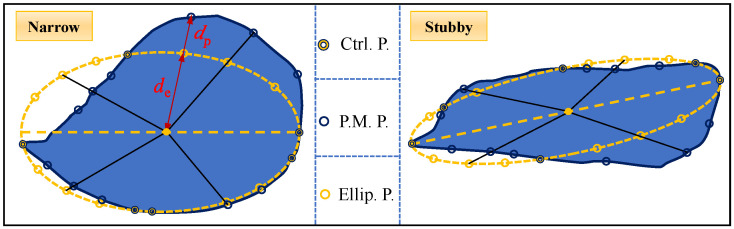
Schematic diagram of the elliptical wrapping method.

First, we need to obtain the elliptic function that is most similar to the granular material; by mapping the set of points on the two-dimensional plane, the points on the perimeter of the granular material can be mapped one by one to the smallest wrapped ellipse of the granular material via the elliptic wrapping method. Two typical types of particle shapes, narrow and stubby, are selected, and the elliptic wrapping method is utilized for the point mapping process. As shown in Figure 3, the Ctrl. P. is the point where the ellipse intersects with the particle, and P.M. P. and Ellip. P. are the points on the corresponding contours of the particle contour and the ellipse contour after discretizing the particle contour and the ellipse contour by the angular homogenization method by *n* parts, respectively (the value of *n* is taken to be 4 in the figure for the sake of visual presentation). The ratio of the connecting lines between the geometric center of the particles and the points on the two contours is used as a parameter for the degree of similarity between the two. The degree of similarity is affected by the lengths of the connecting lines, *d*_e_ and *d*_p_, and the more discrete the particles are when they are evenly divided, the more representative the similarity parameter is. The similarity parameter *λ* can be calculated via the magnitude relationship between *d*_e_ and *d*_p_, as shown in Equation (3).

(3) $$\lambda =\frac{1}{n}\sum_{k=1}^{n}\frac{\min\left(d_{p\left(k\right)},d_{e\left(k\right)}\right)}{\max\left(d_{p\left(k\right)},d_{e\left(k\right)}\right)}$$

Second, the geometric parameters of the ellipse can be obtained after mapping, and the corresponding geometric parameters of the circle can be obtained by utilizing the invariant energy of particle motion. On the basis of the energy law, the particles will slide and rotate at the same time under the action of the dynamic load and generate the corresponding sliding and rotating energies, and the rotating energies of elliptic particles and circular particles, *E*_e_ and *E*_c_, can be obtained via Equation (4) [40].

(4) $$\left\{\begin{array}{l} E_{e}=\frac{1}{2}\left[m{v_{p}}^{2}+\frac{1}{4}m\omega^{2}{\left(a^{4}+b^{4}+4a^{2}b^{2}+{\left(a^{2}-b^{2}\right)}^{2}\right)}^{0.5}\right]\\ E_{c}=\frac{1}{2}\left(m{v_{p}}^{2}+\frac{1}{2}m\omega^{2}r^{2}\right) \end{array}\right.$$

where *m* is the particle mass, kg; *v*_p_ is the particle slip rate, m/s; *a* and *b* are the lengths of the long and short axes of the elliptical particles, mm; *ω* is the angle of rotation of the particles; and *r* is the length of the radius of the circular particles, mm. The energy of motion of the transformed circular particles must be the same as the energy of motion before the transformation, i.e., *E*_e_ = *E*_c_. Therefore, the transformed circular radius *r* can be obtained via Equation (4), as expressed in Equation (5).

(5) r=22a4+b4+4a2b2+a2−b2214

Finally, when the particles are transformed from irregular particles to regular circles, it is necessary to ensure that the particle density remains unchanged, which can be achieved by changing the particle density, as shown in Equation (6).

(6) $$\frac{\rho_{\mathrm{eff}}}{\rho_{p}}=\frac{2s_{\mathrm{own}}}{\pi }{\left[a^{4}+b^{4}+4a^{2}b^{2}+{\left(a^{2}-b^{2}\right)}^{2}\right]}^{-0.5}$$

where *ρ*_eff_ is the converted effective density and *ρ*_p_ is the initial density of the particles, kg/m^3^.

Through the above analysis, we believe that two conditions must be satisfied in the transformation of granular materials from irregular shapes to regular circles, i.e., conservation of motion energy and equal density. The conservation of energy of motion ensures that the energy will not change because of the difference in particle shape when considering the rotation of the particles, and the condition of equal density ensures that the mass of the particles will not change because of the change in area when the particles are converted to other shapes, which makes clear the influence of the shapes of the particles on the particles during the process of particle rotation from the point of view of the intrinsic mechanism.

## 3. Effect of Particle Rotation on Shear Damage

**Figure 4:**
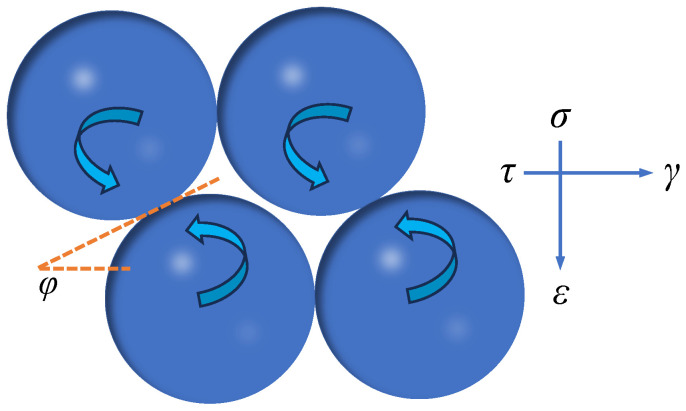
Schematic of particle rotation.

It is commonly accepted in the field of geotechnics that granular materials produce mutual occlusion between particles during shear. When a granular system produces shear damage along the shear plane, the occluded particles must cross or shear neighboring particles, destroying the original soil structure and creating shear damage. As the shear displacement increases, the strength of the system is expressed as the friction strength between discrete particles. However, the soil particle system consists of different particles contacting and combining with each other, and according to the laws of thermodynamics and conservation of capacity, the work performed by the external load consists of two parts: the rotational dissipated energy and the sliding dissipated energy of the particles. Therefore, the sum of the energy required to overcome the sliding and rotating energy of the particles is required for the particle system to undergo shear damage, and the sliding and rotating energies required for particle damage are explored separately from a fine-grained point of view, as shown in Figure 4.

The tangential strain of particles under loading consists of slip strain and rotational strain, and the positive and tangential strains of particles can be expressed by Equation (7) [41].

(7) $$\left\{\begin{array}{l} \varepsilon =\frac{\mu \omega \sin\varphi }{\left(2-2\mu \right)\cos^{2}\varphi }\\ \gamma =\frac{\omega \sec\varphi }{2-2\mu } \end{array}\right.$$

where *ε* and *γ* are the positive and tangential strains, respectively; *μ* is the ratio of the slip tangential strain to the total tangential strain; and *φ* is the angle between the tangent line of the particles and the horizontal direction, which is related to the particle arrangement.

The energy dissipation of a system of particles in a unit volume satisfies Equation (8), i.e., the energy dissipated by the particles is equal to the work performed by the shear stress [35].

(8) $$\frac{\sigma \mu \omega \sin\varphi }{\left(2-2\mu \right)\cos^{2}\varphi }+\frac{\tau \omega \sec\varphi }{2-2\mu }=\frac{\rho_{p}}{\rho_{\mathrm{eff}}}\int \tau_{h}d\gamma_{h}$$

where *τ*_h_ is the macroscopic shear stress, kPa, and *γ*_h_ is the macroscopic shear strain.

The particulate system is formed by the arrangement and combination of the number of the particles and the different sizes of the particles, as determined on the macroscopic scale, which can be characterized as a network composed of Voronoi cytosolic elements by the Voronoi cytosolic element model [42], based on the particulate material.

There are three modes, namely, sliding, rotating, and no contact between particles, and there is actually more than one contact surface for each single particle, which means that the sliding energy of a particle is provided by the forces on all sliding-contact surfaces of the particle, whereas the rotating energy of a particle is similarly provided by the forces on all rotating-contact surfaces of the particle. On the basis of an analysis of the particle-related material in the literature [43], the average particle-to-particle contact area *A* and the average number of contacts *q* can be obtained, as shown in Equation (9).

(9) $$\left\{\begin{array}{l} A=\frac{\pi r^{2}\Delta \psi }{6}\\ q=\frac{1}{r^{3}\cos\theta } \end{array}\right.$$

where Δ*ψ* is the change in porosity during shear, and *θ* is the shear expansion angle of the granular material.

On the basis of the physical and geometrical equations describing the relationships between the particulate materials, we can obtain another expression for the energy dissipated by the particles per unit volume, *E*_d_, as shown in Equation (10).

(10) $$E_{d}=\frac{\tau \pi \Delta \psi }{6\cos\theta }\left[\frac{\omega \sec\varphi }{2-2\mu }-\frac{\omega \left(1-\cos\theta \right)}{2\cos\theta }\right]$$

The expressions for the dissipated energy of the granular material given separately in Equations (8) and (10) are given together in Equation (11).

(11) $$\frac{\sigma \mu \omega \sin\varphi }{\left(2-2\mu \right)\cos^{2}\varphi }+\frac{\tau \omega \sec\varphi }{2-2\mu }=\frac{\tau \pi \Delta \psi }{6\cos\theta }\left[\frac{\omega \sec\varphi }{2-2\mu }-\frac{\omega \left(1-\cos\theta \right)}{2\cos\theta }\right]$$

Simplifying Equation (11) yields an equation for the ratio of positive stresses to shear stresses, as shown in Equation (12).

(12) $$\beta =\frac{\tau }{\sigma }=\frac{\mu \sin\varphi }{\left(1-\mu \right)\cos^{2}\varphi }\left[\frac{\pi \Delta \psi \sec\varphi }{6\left(1-\mu \right)\cos\theta }-\frac{\pi \Delta \psi \left(1-\cos\theta \right)}{6\cos^{2}\theta }-\frac{\sec\varphi }{1-\mu }\right]$$

If granular materials such as glass beads and calcareous sand satisfy the nonviscous Coulomb strength criterion on a macroscopic scale, the right-hand side of Equation (12) is expressed as the tangent of the angle of internal friction of the granular material. Typically, we consider the angle of internal friction of a material to be constant, whereas *β* in Equation (12) is affected by multiple factors, which indicates that the difference in shear strength between discrete granular materials and continuous medium materials, i.e., the case of granular materials, does not precisely obey the Coulomb strength criterion.

## 4. Granular Material Shear Test and Result Analysis

Calcareous sand, a widely distributed cohesionless geotechnical granular material in the South China Sea region, has gradually become an indispensable building material in island construction [44]. Unlike land-sourced quartz sand, calcareous-sand particles are a special kind of geotechnical medium formed by the deposition of marine organisms (shells and carcasses), which are characterized by high porosity, irregular shape, and angularity. Silica beads, the upper regular granular earth material counterpart to irregular calcareous sands, are also widely used in engineering and construction. Therefore, the test objects were silica beads and calcareous sand; the former was considered a regular round granular material, the latter was considered an irregular granular material, and the particle sizes were controlled within a single size grading interval. The Civil Engineering Experimental Center of China University of Mining and Technology-Beijing and the Key Laboratory of Environmental Rocks and Engineering Disaster Control of Jiangxi Province supported the development of basic physical–mechanical tests and shear tests for the two types of granular materials. The vertical loads were set to 100 kPa, 200 kPa, and 300 kPa, and the loading rate was held constant at 0.8 mm/min.

The physicomechanical parameters of the two types of granular materials can be obtained via basic determination tests, and the results are shown in Table 2.

**Figure 5:**
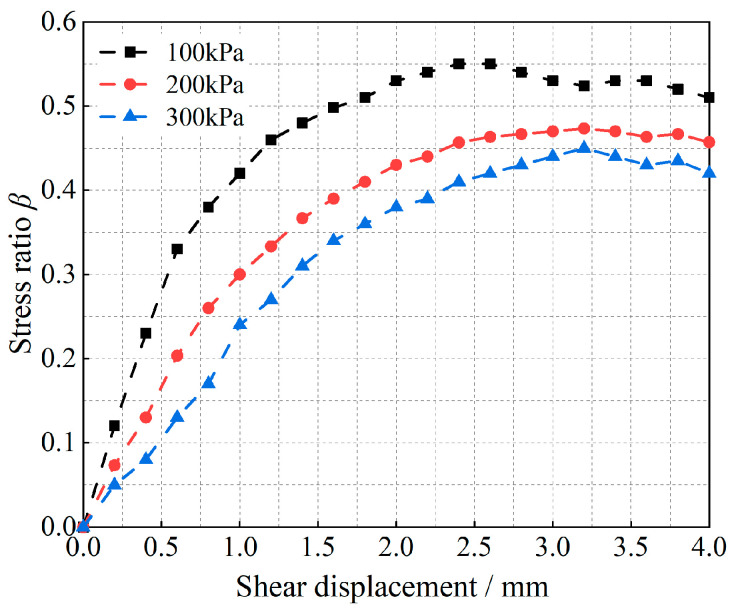
Relationship between the shear-stress-to-positive-stress ratio and the shear displacement of glass beads.

**Figure 6:**
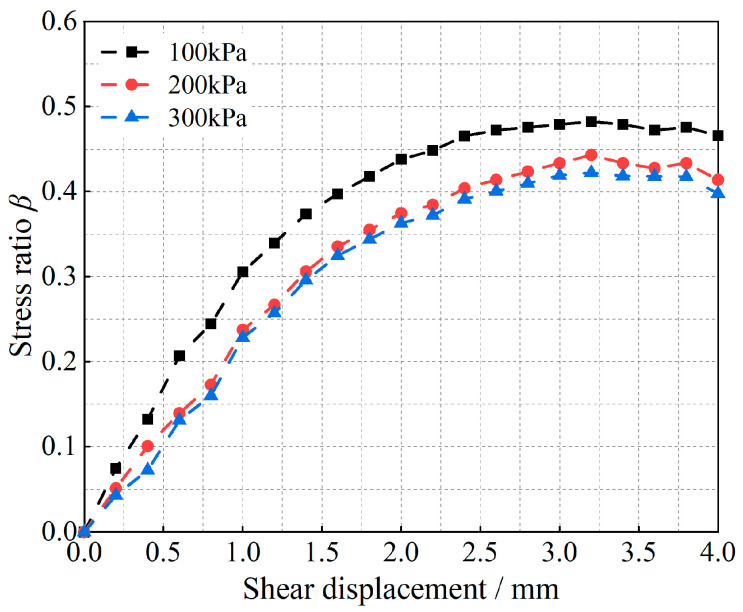
Relationship between the shear-stress-to-positive-stress ratio and the shear displacement of calcareous sand.

Shear tests are carried out on both types of granular materials, and the corresponding shear-damage results are shown in Figure 5 and Figure 6.

As shown in Figure 5 and Figure 6, the curves demonstrate the variation in the stress ratio with shear displacement for glass beads and calcareous sand under different vertical loads, and they can also be regarded as the curves of the variation in the particle shear strength with shear displacement, since the vertical load is kept constant with respect to the stress ratio. Figure 5 shows that the peak shear strength of the glass-bead particles at 100 kPa appears at a shear displacement of 2.5 mm, whereas the peak shear strengths of 200 kPa and 300 kPa appear at shear displacements of 3.2 mm. Additionally, shear vibration oscillations occur after shear damage occurs, and increasing the vertical load reduces the shear displacement required for damage to occur in the particle system. Moreover, observing the change in the slope of the curve in the figure reveals that the curve is almost linear before the shear displacement of 1.4 mm, i.e., the slope is almost unchanged; thereafter, the slope changes with the change in shear displacement, which indicates that the shear modulus of the granular material also changes. The curves of the variation in the shear-stress ratios of calcareous sand for the three vertical loading conditions in Figure 6 show the same increasing pattern, differing only in the magnitude of the increase in the curves and the shear displacements required for the occurrence of shear damage, owing to the differences in the types of materials.

**Figure 7:**
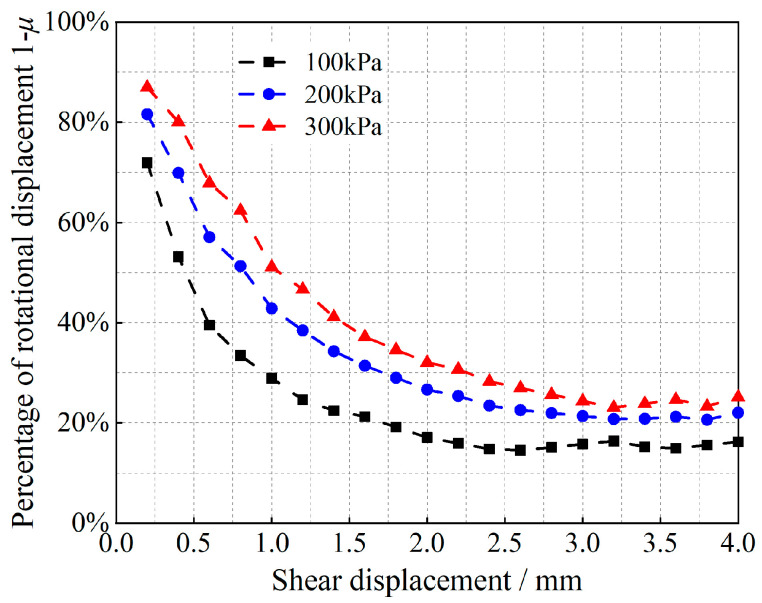
Relationships between the particle rotational properties and the shear displacement of glass beads.

**Figure 8:**
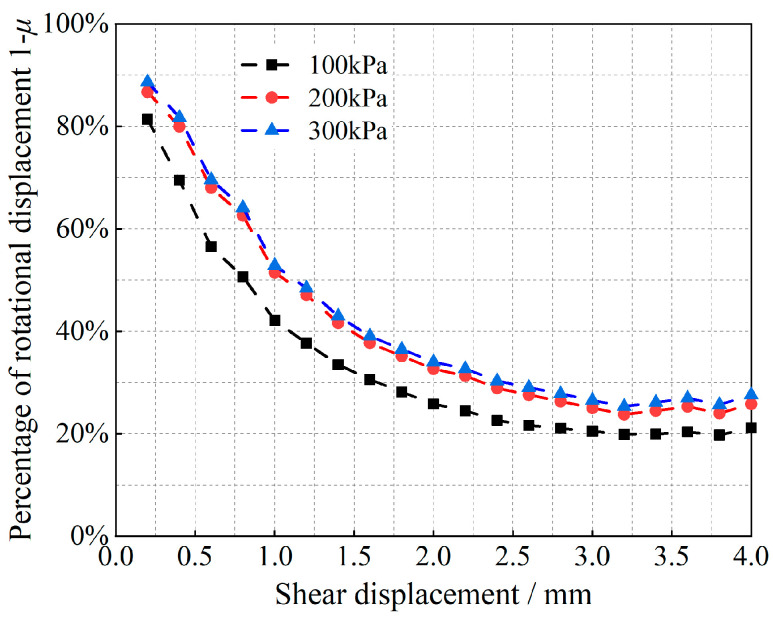
Relationships between the particle rotational properties and the shear displacement of calcareous sand.

**Figure 9:**
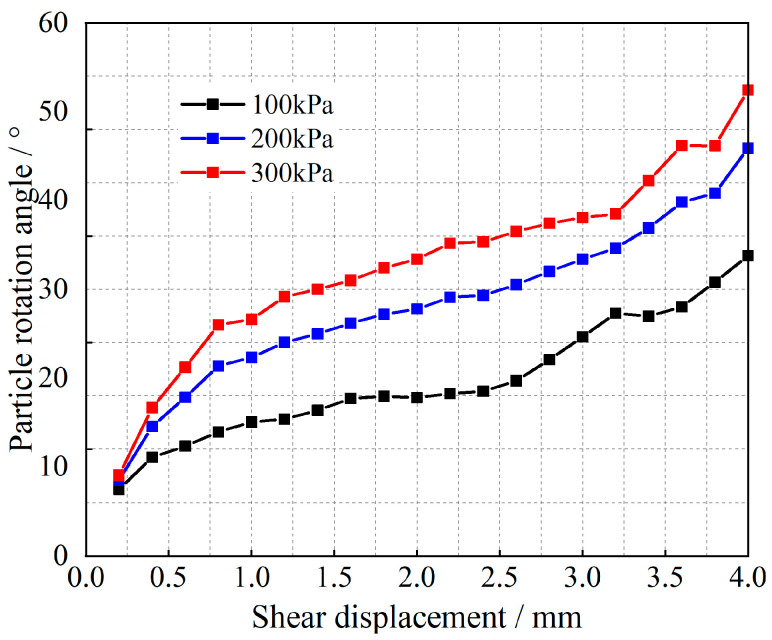
Relationship between the angle of rotation and the shear displacement of glass beads.

**Figure 10:**
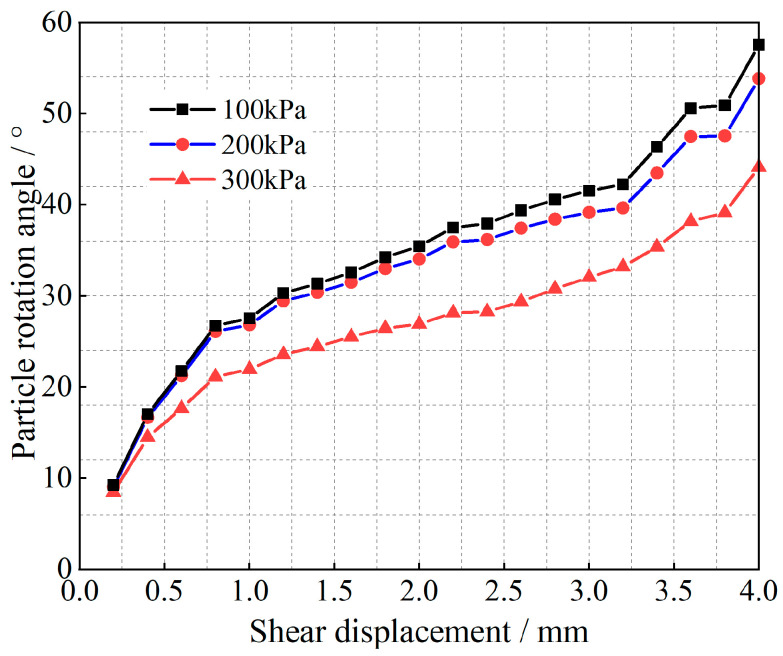
Relationship between the angle of rotation and the shear displacement of calcareous sand.

Equation (12) is a quadratic function of the ratio *μ* of the slip-induced shear displacement to the total shear displacement, and the corresponding (1 − *μ*) is the ratio of the shear displacement due to rotation to the total shear displacement. Tong et al. [35] obtained the angle of rotation of the particles; the results are shown in Figure 7, Figure 8, Figure 9 and Figure 10.

As shown in Figure 7, Figure 8, Figure 9 and Figure 10, at the initial stage of shear action, the percentages of shear displacement caused by the rotation of glass beads and calcareous-sand particles reach 86.9% and 88.7%, respectively, under the 300 kPa vertical load, and the same percentages reach 71.9% and 81.4% at 100 kPa, which indicates that the particle rotation at the initial stage is the main causative factor for the initial shear change. With increasing shear displacement, the percentage of shear displacement caused by particle rotation decreases sharply, and the inflection point of the decrease appears at 1.4 mm of shear displacement. The curve then slowly decreases and remains stable, whereas the shear displacement caused by particle slip simultaneously increases. After shear damage occurs when the shear displacements exceed 2.5 m or 3.2 mm, the percentage of rotationally induced shear displacement decreases to less than 20%. The observation of the cumulative rotational angle of the particles reveals that the inflection point at which the rotational angle increases appears earlier than the inflection point at which the ratio decreases, which is due to the shear displacement growth of the rotation and slip gradually reaching an equilibrium state; thus, the equilibrium state must be broken to exhibit more slip shear, and the corresponding particles undergo rotational weakening. Shear damage occurs under the rotational angles associated with the same oscillatory changes. Observations of the rotational properties under different grades of vertical loads revealed that glass beads are more sensitive to vertical loads than calcareous sand is.

**Figure 11:**
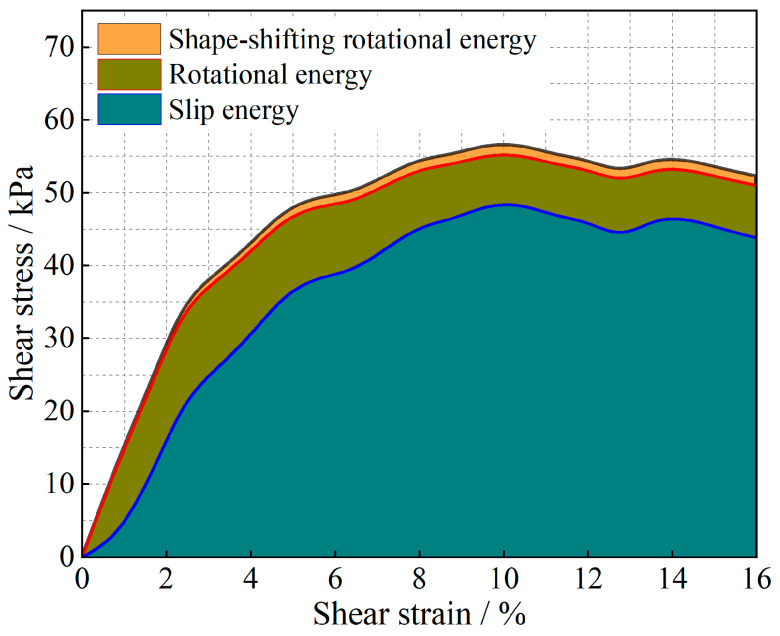
Strain-energy density evolution in glass-bead granular systems (under 100 kPa).

**Figure 12:**
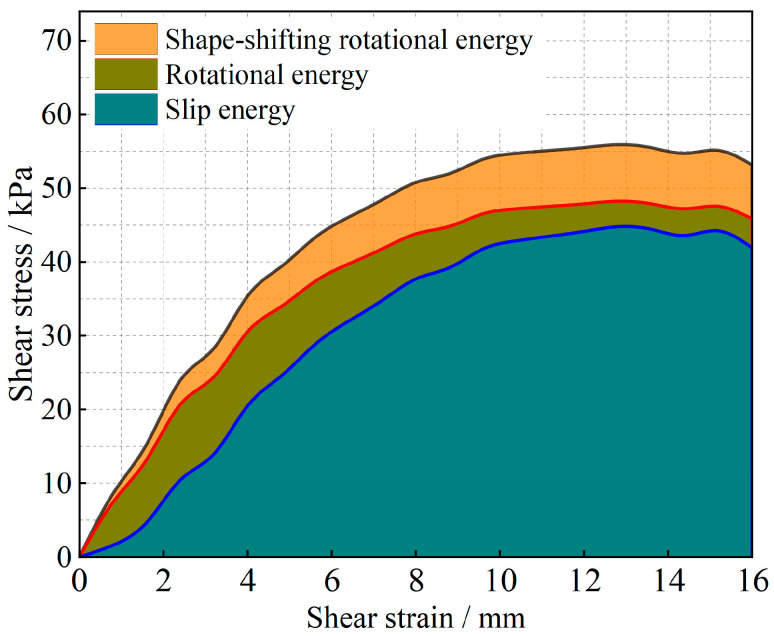
Strain-energy density evolution in calcareous-sand granular systems (under 100 kPa).

**Figure 13:**
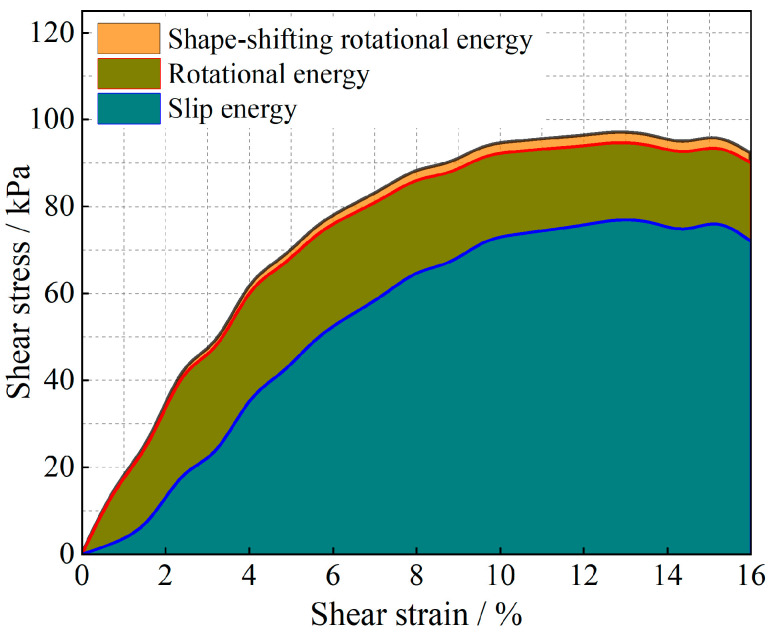
Strain-energy density evolution in glass-bead granular systems (under 200 kPa).

**Figure 14:**
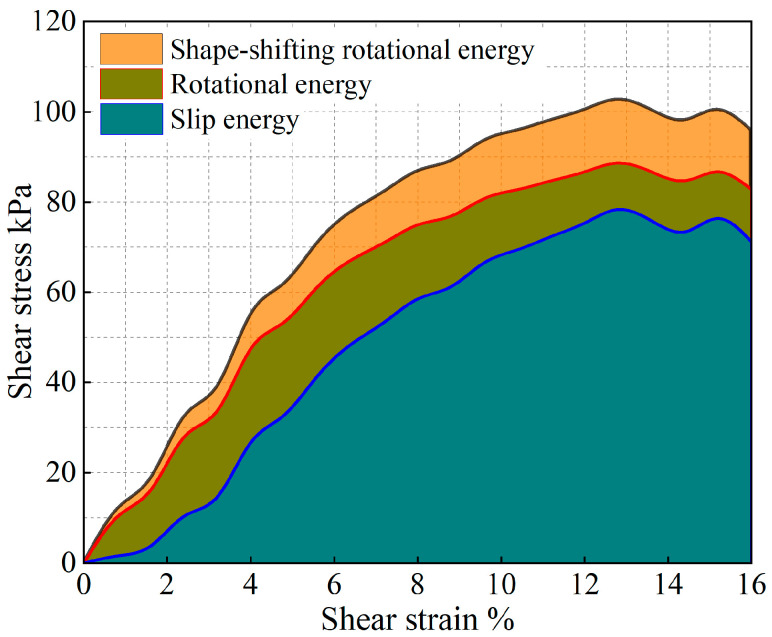
Strain-energy density evolution in calcareous-sand granular systems (under 200 kPa).

**Figure 15:**
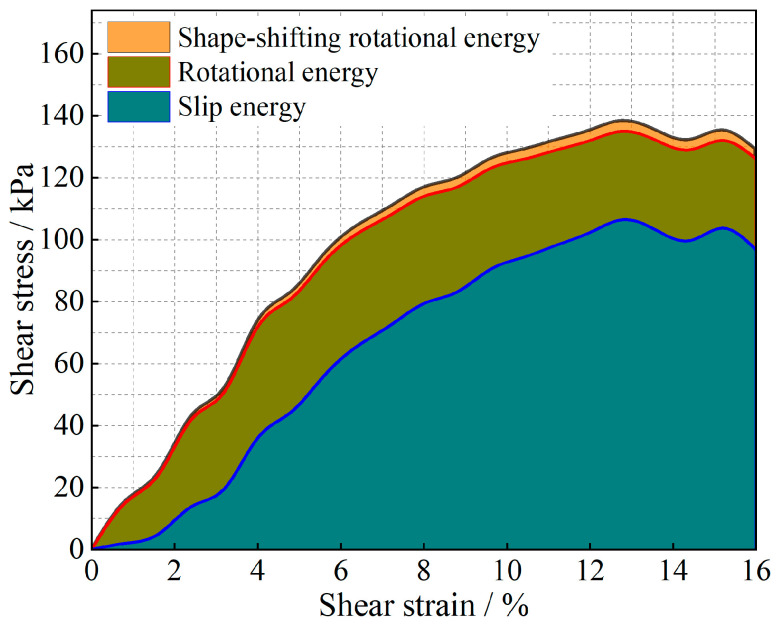
Strain-energy density evolution in glass-bead granular systems (under 300 kPa).

**Figure 16:**
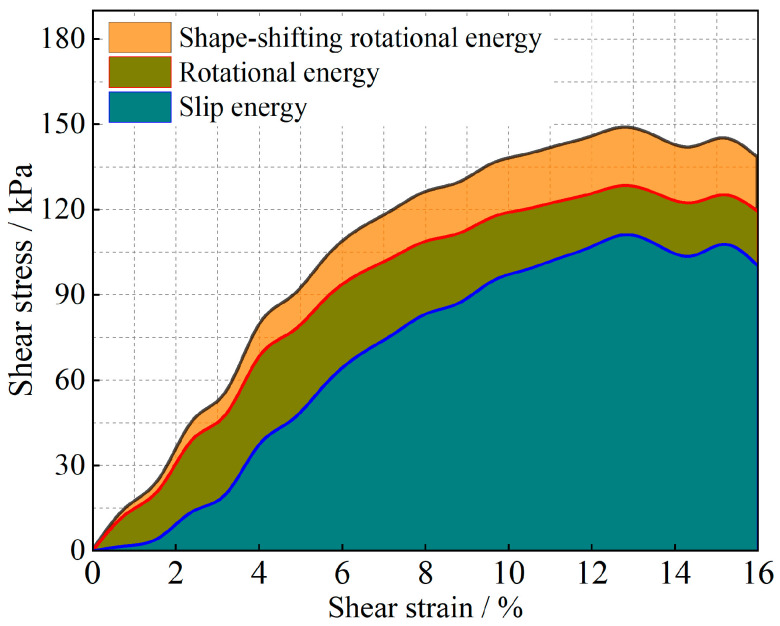
Strain-energy density evolution in calcareous-sand granular systems (under 300 kPa).

The evolution of the particle system during shear is analyzed from an energy point of view, and the results are shown in Figure 11, Figure 12, Figure 13, Figure 14, Figure 15 and Figure 16.

As shown in Figure 11, Figure 12, Figure 13, Figure 14, Figure 15 and Figure 16, the area enclosed by the shear stress-strain curve of the granular material under the action of shear stress is expressed as the energy density of the system, and the work performed by the external load on the system consists of two parts: the sliding-shear energy of the particles (the cyan part) and the rotational-shear energy (the dark yellow part + the orange part), of which the particle rotational energy consists of the conversion from the circular particle. In this case, the total dissipated energy density of the particle system is obtained from the so-called area of the stress-strain curve in the chamber, and the percentages of rotational and sliding energy are calculated from the percentages of rotational versus sliding displacement produced by shear stress. The rotational energy of the particles consists of the converted round particles’ own rotational energy (orange part, referred to as their own rotational energy) and the conversion of irregular particles into round particles’ rotational energy (dark yellow part, referred to as the shape shear energy). Among these values, in the early stage of shear, the particle rotational-shear energy mainly encompasses the work performed by the external force, and with increasing strain, the mobile shear gradually increases and exceeds the rotational-shear energy until it becomes the main dissipation energy, which means that the effect of particle rotation on the shear of the system cannot be ignored, especially as to materials in which the shear action has just occurred. In addition, the rotational energy of the glass-bead particles far exceeds the shape shear energy, whereas the rotational energy of the calcareous-sand particles almost coincides with the natural shear energy, and irregular calcareous sands need more energy for shape transformation than regular glass beads do. When comparing the energy evolution of the particle system under different orthogonal vertical loads, the three energies show completely inconsistent development trends with increasing vertical load, i.e., the amplitude of the particle’s slip shear energy increases, while its proportion decreases; the amplitude of the particle’s own rotational energy increases, while its proportion increases; the amplitude of the particle shape rotational energy is almost unchanged, and its proportion decreases. This shows that the effect of vertical loading on the rotational shear of particles is significantly greater than that of sliding shear, and the effect is mainly concentrated in the rotational process rather than the shape transformation process.

**Figure 17:**
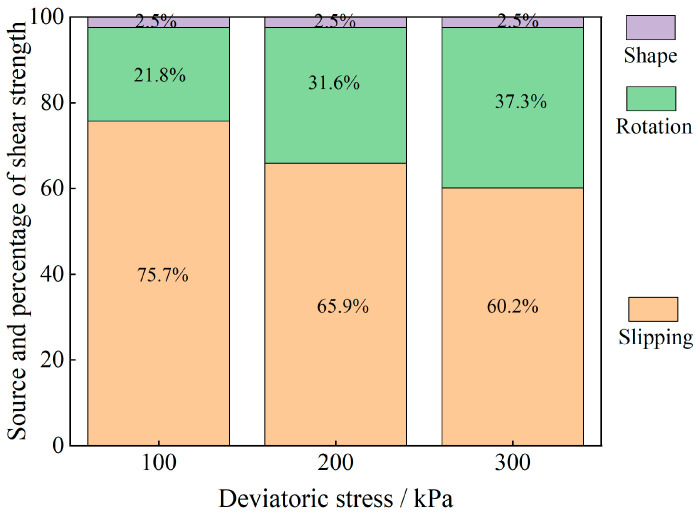
Sources and percentages of the shear strengths of the glass-bead granular materials (after averaging).

**Figure 18:**
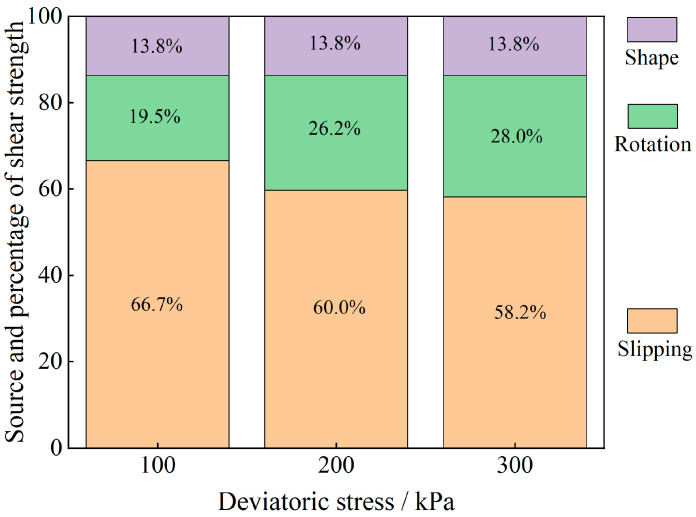
Sources and percentages of the shear strengths of the calcareous-sand granular materials (after averaging).

Owing to the above analysis, we consider that the energy change of soil particles in the shear process is caused completely by particle motion, and this energy change caused by particle motion is composed of two parts: sliding energy and rotational energy. To analyze the proportion of energy changes caused by different modes of motion in the whole process of damage to soil particles from the beginning of shear to the condition of shear, we carry out the analysis by using the stress-strain curves in the shear test and the energy equations in Equations (10)–(12). The results are shown in Figure 17 and Figure 18. The shape-induced energy change is a rotational energy that is provided by the conservation of energy maintained during the conversion of irregular particles to regular particles in particle rotation engineering. This means that the closer the particles are to a regular round shape, the closer the percentage of shape-induced energy change is to 0.

The energy evolution history of different materials under different bias stresses is obtained through stress-strain curves, and the sliding-induced energy and rotation-induced energy are calculated by using the scaling factor μ. Finally, on the basis of the law of energy conservation, the shape-induced energy within the change in rotational energy is obtained as a proportion of the rotational energy and is converted to a proportion of the total energy. Through the schematic diagram of the percentages of energy change caused by different factors in Figure 17 and Figure 18, we find that the shear strength of the granular material comes not only from overcoming the sliding friction resistance between particles but also from the rotational friction resistance, and the percentage of rotation-induced energy change in the calcareous-sand particles with a greater degree of irregularity of the soil particles is far greater than that in the regular silica beads, which is mainly because the irregular shape of the calcareous sand greatly affects the rotational properties of the particles. The main reason is that the shape of irregular calcareous sand greatly affects the rotational characteristics of the particles. As the deviatoric stress increases, the percentage of sliding-shear strength of the particles gradually decreases, whereas the percentage of rotational-shear strength increases. Moreover, the percentage of rotational-shear strength of calcareous-sand granular materials is significantly greater than that of glass beads because the irregularity of calcareous-sand granules requires more energy to be expended when rotation occurs, i.e., more shear strength is provided. This also suggests that if the effect of rotation on the shear properties of calcareous-sand materials is not considered and they are considered to experience sliding shear only, the shear strength of the material will be substantially overestimated.

Several interesting conclusions can be drawn from the above analysis of the shear properties of granular materials. First, the two types of noncohesive granules, glass beads and calcareous sand, do not fully comply with the Coulomb strength criterion. Second, the shear modulus of granular materials changes when a certain shear displacement occurs and when the particles are not sheared, not after shear damage occurs, as is often assumed. The shear damage of granular materials is initially caused by particle rotation and later by particle sliding. Finally, ignoring irregular particle shapes and analyzing them directly as round particles leads to energy non-conservation between the system energy and the work performed by external forces. A series of analyses has revealed that simply treating a granular material, such as glass beads and calcareous sand, as a continuous medium results in flawed results and cannot explain the shear phenomena described above.

## 5. Conclusions

(1) Shape parameters are introduced to irregular granular materials to analyze the overall regularity of the particles, and in particle shape conversion, which is based on the conservation of energy of motion and density, two conditions are considered to ensure that the energy and mass of the particles in the process of rotation do not change because of the change in shape of the particles. Moreover, in the analysis of their simplicity, the limitations of direct assumptions are overcome. By performing shear tests on glass beads and calcareous sand, it was found that, unlike continuous media materials, granular materials do not fully comply with the Coulomb strength criterion, and the shear-stress ratio of granular materials first linearly increases with shear displacement and then steadily changes until granular shear damage occurs.

(2) The energy evolution of granular material systems is affected by both particle sliding and particle rotation, and the particle shape changes the energy evolution by affecting the particle’s kinematic properties. The work performed by the external force in the early stage of shear is mainly converted into particle rotational-shear energy, whereas in the later stage, it is mainly converted into particle sliding-shear energy, and the sensitivity of the particle rotational energy to the vertical load is much greater than that of the particle sliding energy. Compared with irregular granular materials, regular granular materials are more prone to rotational behavior, which results in a maximum of 37.3% of the energy being lost due to granular rotation during shear, and the percentage increases with increasing enclosing pressure.

(3) Because the geometry of the irregular particle material is not conducive to particle movement, its conversion to regular particles in the conversion of shear energy will have a significant effect on the determinations, compared to regular particles, because the former’s ratio of all shear energy can reach a maximum of 13.8%, and the ratio of its share of the rotational energy can reach a maximum of 70.8%. The shear energy evolution, as determined by the shapes of the particles and the relevant rotational characteristics, cannot simply be ignored. Redundancy considerations and their nonlinear relationship with bias stress should be considered when analyzing particle shear strength in engineering.

## Figures and Tables

**Table 1:** Calculated results of the shape parameters of granular materials, containing typical and average values.

Particle Types	*AR*	*C*	*S*	*OR*
Glass beads (Typ)	0.95	0.99	0.99	0.96
Glass beads (Avg)	0.97	0.96	0.97	0.97
Glass beads (Range)	[0.95–0.98]	[0.95–0.98]	[0.95–0.98]	[0.95–0.98]
Calcareous sand (Typ)	0.55	0.94	0.83	0.77
Calcareous sand (Avg)	0.63	0.92	0.81	0.79
Calcareous sand (Range)	[0.52–0.77]	[0.91–0.96]	[0.79–0.82]	[0.76–0.80]

**Table 2:** Selected physical and mechanical properties of the glass beads and calcareous sand.

Parameters	Glass Bead	Calcareous Sand	Parameters	Glass Bead	Calcareous Sand
Particle size range *d*/mm	0.20–0.26	0.37–0.89	Equivalent particle size *r*/mm	0.22	0.61
Initial density *ρ*_p_/kg·m^−3^	2647	2728	Effective density *ρ*_eff_/kg·m^−3^	2582	2352.9
Dilation angle *θ/*°	5.6	28.3	Porosity *ψ*/-	0.37	0.55

## Data Availability

The raw data supporting the conclusions of this article will be made available by the authors on request.
